# FACSCanto II and LSRFortessa flow cytometer instruments can be synchronized utilizing single‐fluorochrome–conjugated surface‐dyed beads for standardized immunophenotyping

**DOI:** 10.1002/jcla.23361

**Published:** 2020-05-20

**Authors:** Annelisa M. Cornel, Christine A. J. van der Burght, Stefan Nierkens, Jeroen F. van Velzen

**Affiliations:** ^1^ Center for Translational Immunology University Medical Center Utrecht Utrecht University Utrecht The Netherlands

**Keywords:** FACSCanto II, flow cytometry, immunophenotyping, LSRFortessa, multicenter comparability, standardization

## Abstract

**Background:**

Multiparameter flow cytometry is the preferred method to determine immunophenotypic features of cells present in a wide variety of sample types. Standardization is key to avoid inconsistencies and subjectivity of interpretations between clinical diagnostic laboratories. Among these standardization requirements, synchronization between different flow cytometer instruments is indispensable to obtain comparable results. This study aimed to investigate whether two widely used flow cytometers, the FACSCanto II and LSRFortessa, can be effectively synchronized utilizing calibration bead–based synchronization.

**Method:**

Two FACSCanto II and two LSRFortessa flow cytometers were synchronized with both multicolor hard‐dyed and single‐fluorochrome–conjugated surface‐dyed beads according to the manufacturer's instructions. Cell staining was performed on five whole‐blood samples obtained from healthy controls and were analyzed upon synchronization with the respective synchronization protocols.

**Results:**

Comparability criteria (defined as <15% deviation from the reference instrument) were met with both bead sets when synchronizing different FACSCanto II or LSRFortessa instruments. However, we observed that the criteria could not be met when synchronizing FACSCanto II with LSRFortessa instruments with multicolor hard‐dyed beads. By utilizing single‐fluorochrome–conjugated surface‐dyed beads to determine and adjust PMT voltages, the accepted comparability criteria were successfully met. The protocol has been validated using five different eight‐parameter stained samples.

**Conclusion:**

We show that FACSCanto II and LSRFortessa instruments can effectively be synchronized using single‐fluorochrome–conjugated surface‐dyed beads in case deviation criteria cannot be met using multicolor hard‐dyed beads. Synchronization with single‐fluorochrome–conjugated surface‐dyed beads results in decreased deviations between instruments, allowing comparability criteria to become stricter.

## 1 INTRODUCTION

In the past decades, precise identification and increasingly complex immunophenotyping of neoplastic hematopoietic cells in a variety of tissues have become feasible by the advances in multiparameter flow cytometry technology.^1^, ^2^ Standardization of these complex panel measurements is key to avoid inconsistencies and subjectivity of interpretations between clinical diagnostic laboratories.^3^, ^4^, ^5^, ^6^, ^7^, ^8^, ^9^, ^10^, ^11^, ^12^, ^13^, ^14^, ^15^, ^16^, ^17^, ^18^ The recommendations and guidelines reported by experts in the field can be roughly divided into two main topics: (1) standardization of reagent use and sample preparation and (2) standardization of the acquired results on different instruments (from now on referred to as synchronization). Synchronization of flow cytometers is described in many variations, ranging from protocols synchronizing FSC/SSC characteristics^10^, ^11^, ^12^ to protocols synchronizing multiple‐color flow cytometry.^3^, ^4^, ^5^, ^6^, ^16^, ^18^ Even though these protocols vary in utilized standardization methods, they all agree on their main goal to achieve uniform and comparable instrument sensitivity levels, reproducible percentages, and expression patterns on different instruments.

Synchronizing instruments in different laboratories and different countries makes the use of biological samples impractical. As a result, a variety of beads have been developed which can be utilized to synchronize multiple instruments to approximately the same conditions. Available beads can be roughly divided into two categories: hard‐dyed beads and surface‐dyed beads. Hard‐dyed beads have incorporated dyes in the polymer matrix, whereas surface‐dyed beads are covalently linked with fluorochromes, thereby more closely resembling the biological situation.^15^ Hard‐dyed beads have a fluorochrome stability of at least two years, which is their main advantage. In contrast, surface‐dyed beads are highly thermally and photolytically unstable. A clear disadvantage of hard‐dyed beads over surface‐dyed beads is that the dyes incorporated in hard‐dyed beads merely share optical properties, but are not spectrally equivalent to the fluorochromes utilized in immunophenotyping of biological samples.

Synchronization of instruments utilizing multicolor hard‐dyed beads is a widely accepted synchronization strategy, as, for instance, described in the EuroFlow standard operating procedure (SOP)^3^ and in the ONE study.^4^ The recommendation is to first determine the mean fluorescence intensity (MFI) on a reference flow cytometer using multicolor hard‐dyed beads. Subsequently, the beads are acquired on the flow cytometer to be matched and the photomultiplier tube (PMT) voltages are adjusted to meet a comparable MFI as measured on the reference flow cytometer. The acceptable comparability criteria are set on a <15% deviation from the reference instrument MFI. Synchronization was proven to be effective on the four 8‐color flow cytometry instruments that were available when the EuroFlow project started in 2006 (FACSCanto II, FACSAria, LSR II, and CyAn ADP)^3^ as well as between Navios flow cytometers.^4^ All four instruments have a three‐laser‐line configuration, with blue (488 nm), red (633 or 635 nm), and violet (405 or 407 nm) lasers.

However, as technology evolved, several new instruments have emerged which are equipped with a four (or even more)‐laser‐line configuration, like the LSRFortessa. Utilizing these instruments allows for measurement of more than double the number of parameters within one sample. This type of flow cytometer instruments will increasingly be used in centers to be able to keep up with the majorly increasing amount of knowledge gained about types of neoplastic hematopoietic malignancies and treatment parameters. In an effort to synchronize multiple FACSCanto II and LSRFortessa instruments, we observed that acceptability criteria (<15% deviation) could not be met with multicolor hard‐dyed beads. We therefore compared the level of deviation between the multicolor hard‐dyed bead synchronization protocol and a method using single‐fluorochrome–conjugated surface‐dyed beads for synchronization of the FACSCanto II and the LSRFortessa (equipped with blue (488 nm), red (640 nm), violet (405 nm), or UV (355 nm) lasers) analyzing eight PMTs. We here report that synchronization using single‐fluorochrome–conjugated surface‐dyed beads results in less deviation than the use of multicolor hard‐dyed beads to determine and adjust PMT voltages. The protocol has been validated using five different eight‐parameter stained samples.

## 2 MATERIAL AND METHODS

### 2.1 Flow cytometer specifications

Two 3‐laser FACSCanto II (BD Biosciences, Eysins, Switzerland), equipped with a 405‐nm, 488‐nm, and 633‐nM laser, and two 4‐laser LSRFortessa (BD Biosciences) cytometers, equipped with a 405, 488, 561‐nm, and 633‐nm laser, were used for these experiments. All instruments had matching filter configurations: a 450/50 and 510/50 BP filter for the 405‐nM laser, a 660/20 and 780/60 BP filter for the 633‐nM laser, and a 530/30 BP and 670 LP filter for the 488‐nm laser. PE and PE‐tandem labels are differently excited on the FACSCanto II (488‐nm laser) and the LSRFortessa (561‐nm laser). Detection was the same between instruments (585/42 and 780/60 BP filters). Furthermore, FACSCanto II laser power was 405 nM ± 25 mW, 488 nm ± 15 mW, and 633 nM ± 15 mW, whereas LSRFortessa laser power was 405 nm ± 40 mW, 488 nm ± 40 mW, 633 nm ± 40 mW, and 561 nm ± 40 mW.

CS&T beads (BD Biosciences, CE‐IVD for FACSCanto instruments (catalog 662413) and research grade for LSRFortessa instruments [catalog 650622]) were used to check the performance of the flow cytometer and verify optical path and stream flow. This procedure enables controlled standardized results and allows the determination of long‐term drifts and incidental changes within the flow cytometer. CS&T beads were measured before each analysis to verify optimal performance of the flow cytometer. No changes were observed which could affect the results.

### 2.2 Experimental setup of synchronization

First, eight‐peak Sphero™ Rainbow bead calibration particles (Spherotech [catalog RCP‐30‐5A]) were used to perform synchronization between flow cytometers.^3^ In short, the multicolor hard‐dyed calibration beads were used to determine the MFI on a reference flow cytometer. Subsequently, beads were measured on the flow cytometer to be matched and each of the eight PMT voltages was adjusted to meet a comparable MFI as measured on the reference cytometer.

Subsequently, the potential of single‐fluorochrome–conjugated surface‐dyed BD™ FC beads (BD Biosciences [catalog 658621]) was tested to adjust PMT voltages. Each tube contained both negative polystyrene beads and beads coupled to one specific fluorochrome. In this way, every PMT voltage adjustment is performed with a separate tube containing beads with the fluorochrome of interest. PMT voltage adjustment was performed according to the above‐described procedure. The acceptable comparability criteria are set on a <15% deviation from the reference instrument MFI.^3^

### 2.3 Compensation

BD™ CompBeads particles (BD Biosciences) were used on all instruments to compensate for spectral overlap according to the manufacturer's instructions. A mixture of anti‐mouse Ig‐κ‐conjugated and non‐conjugated negative control CompBeads was made. Fluorochrome‐conjugated mouse κ‐light chain–bearing immunoglobulin will bind to the Ig‐κ‐conjugated beads, and the negative and positive peaks were subsequently used to determine compensation percentages. Measurement of these single‐antibody–labeled beads was repeated for every fluorochrome‐conjugated antibody of interest. Single‐fluorochrome–conjugated surface‐dyed peripheral blood mononuclear cell (PBMC) samples were measured to verify the compensation matrix.

### 2.4 Sample preparation

Cell staining was performed on five EDTA‐containing whole‐blood samples obtained from healthy donors who gave their informed consent to participate in this study. Whole‐blood samples containing 1 × 10^6^ cells were lysed by a 15‐minute incubation with 1× BD Pharm Lyse™ solution at room temperature (BD Biosciences [catalog 555899]) and subsequently washed twice with PBS/HSA (0.5%) (1800 rpm, 10 minutes). Cells were incubated with titrated amounts of monoclonal antibodies directed against CD2 FITC (clone S5.2), CD3 PerCP‐Cy5.5 (clone SK7), CD8 APC (clone SK1) and CD4 Pacific Blue (PB) (clone RPA‐T4) (all from BD Biosciences), CD19 APC‐A750 (clone J3‐119), CD7 PE (clone 8H8.1), CD14 PE‐Cy7 (clone RMO52) (all from Beckman Coulter), and CD45 Pacific Orange (PO) (clone HI30; Life Technologies) in a total staining volume of 80 µL. Samples were incubated for 15 minutes at room temperature in the dark, washed (500 *g*, 10 minutes), resuspended in 300 µL PBS/HSA (0.5%), and analyzed on all instruments in a 30‐minute time frame.

### 2.5 Sample analysis

**FIGURE 1:**
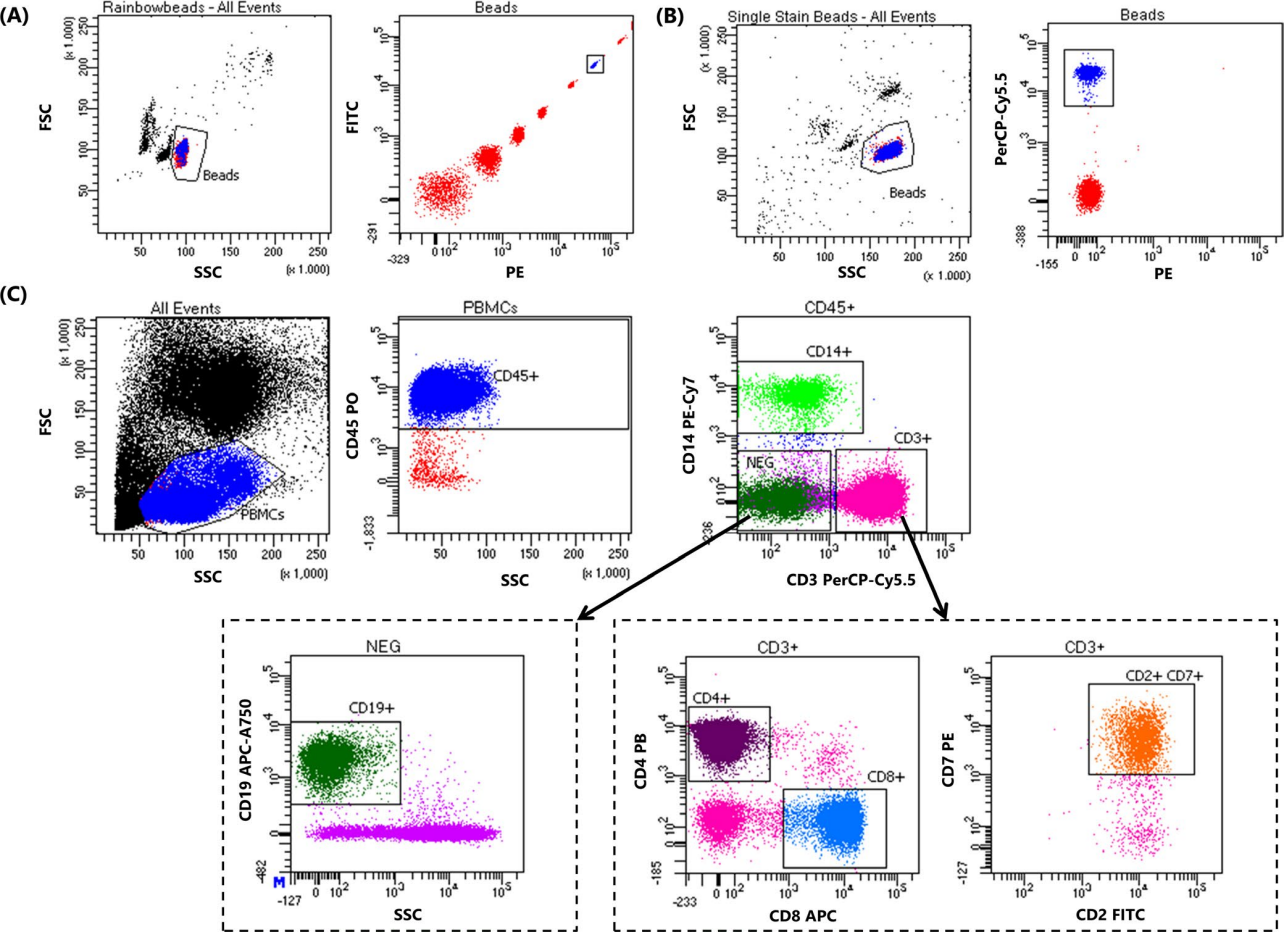
Synchronization gating strategies. A, Multicolor hard‐dyed bead calibration. Bead population is identified based on FSC/SSC characteristics, after which the sixth rainbow particle peak is identified based on emission characteristics and used to match PMT voltages. B, Single‐fluorochrome–conjugated surface‐dyed fluorescently labeled bead calibration. Bead population is identified based on FSC/SSC and emission characteristics in the channel of interest and used to adjust PMT voltages. C, Gating strategy of eight‐parameter stained whole‐blood samples. Lymphocytes were identified based on FSC/SSC and CD45 (PO) expression. From the CD45 + population, B cells were identified based on the absence of CD14 (PE‐Cy7) and CD3 (PerCP‐Cy5.5) and the presence of CD19 (APC‐A750). Monocytes were identified based on CD14 expression, whereas T cells were identified based on CD3, CD2 (FITC), and CD7 (PE), and either CD4 (PB) or CD8 (APC) expression

The eight‐peak Sphero™ Rainbow bead calibration particles were identified based on FSC/SSC characteristics, after which the eight different bead populations can be distinguished based on emission characteristics (Figure 1A). A gate was drawn which included the sixth emission peak (Figure 1A), after which MFIs of all PMTs were determined on one instrument. The obtained reference MFIs were subsequently used as target MFIs for all other instruments. Single‐fluorochrome–conjugated surface‐dyed BD™ FC beads were identified based on FSC/SSC characteristics, after which a negative and positive emission population can be distinguished in the channel of the single PMTs of interest (Figure 1B). The obtained reference MFI was subsequently used as a target for all other instrument. This was repeated for every PMT of interest.

To be able to assess the efficiency of the synchronization protocols, whole‐blood samples were subsequently analyzed on two FACSCanto II and two LSRFortessa flow cytometers. The gating strategy is shown in Figure 1C to be able to identify which cell populations are utilized to compare MFIs between instruments. The percentage deviation from the reference MFI (% dev. from ref.) was calculated using the following equation:

$$\%\;\text{dev}.\;\text{from}\;\text{ref}.\;=\;\frac{\text{obtained}\;\text{MFI}\;-\;\text{reference}\;\text{MFI}}{\text{reference}\;\text{MFI}}.$$

Percentages and MFI of all differently emitting populations were compared and expressed as mean ± standard deviation (SD). Data were analyzed using FACS DIVA version 8.0.1 (BD Biosciences).

## 3 RESULTS

### 3.1 Synchronizing FACSCanto II and LSRFortessa with multicolor hard‐dyed bead synchronization

**FIGURE 2:**
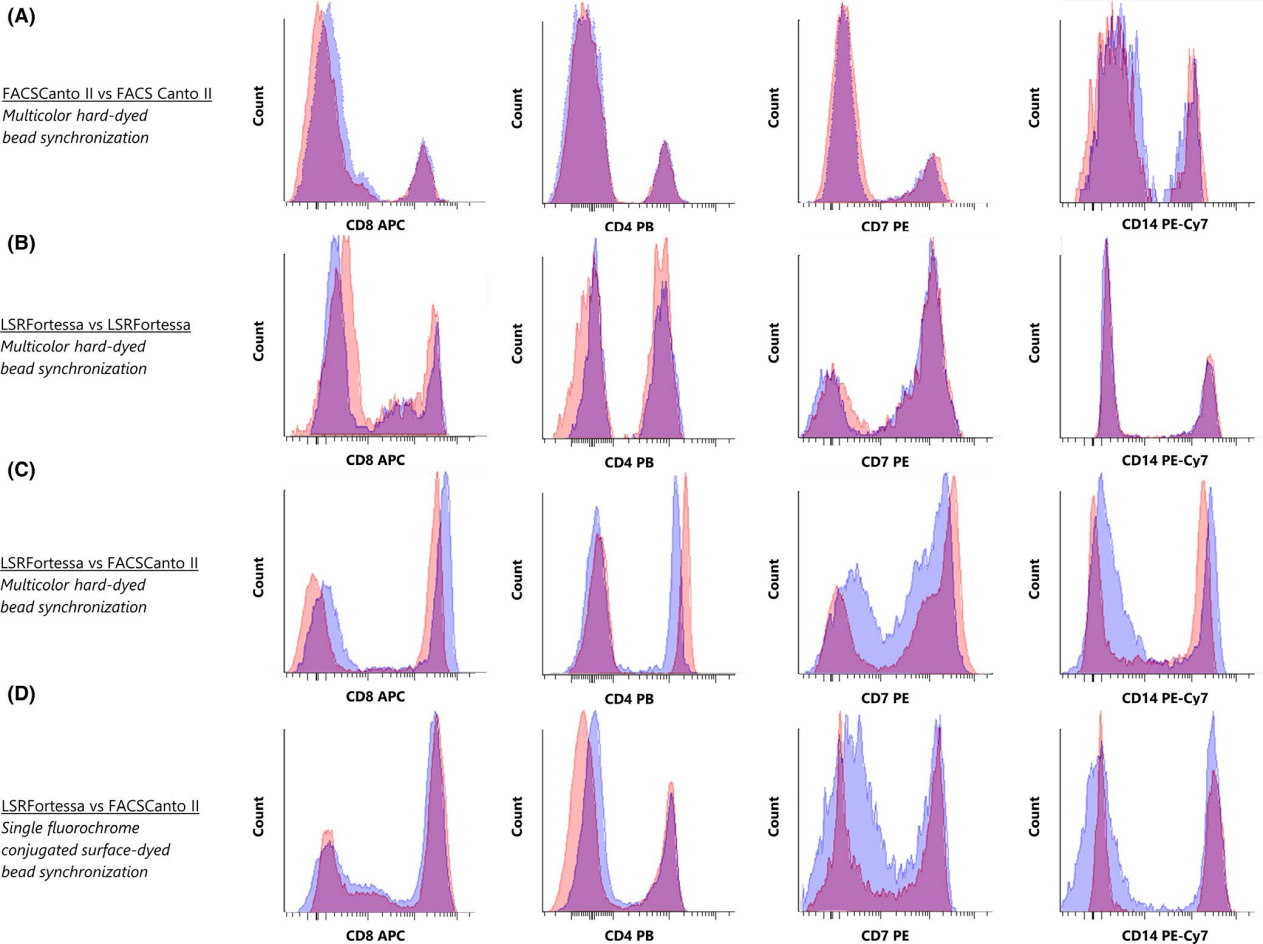
Expression patterns of four parameters between synchronized flow cytometer instruments. Representative expression patterns are shown in overlay histogram plots. Four of the eight analyzed parameters are shown (from left to right: APC—PB—PE—PE‐Cy7). A, FACSCanto II vs FACSCanto II using multicolor hard‐dyed bead calibration. B, LSRFortessa vs LSRFortessa using multicolor hard‐dyed bead calibration. C, FACSCanto II vs LSRFortessa using multicolor hard‐dyed bead calibration. D, FACSCanto II vs LSRFortessa using single‐fluorochrome–conjugated surface‐dyed fluorescently labeled bead calibration

In the first part of this study, we investigated whether the multicolor hard‐dyed bead synchronizing protocol can be extended to include the LSRFortessa. Two FACSCanto II and two LSRFortessa flow cytometers were synchronized using the multicolor hard‐dyed bead protocol (Figure 1A).^3^ Compensation of spectral overlap was applied as described. Subsequently, to assess synchronization efficiency, five different eight‐parameter stained whole‐blood samples were analyzed on all flow cytometers, after which percentages of FACSCanto‐FACSCanto, LSRFortessa‐LSRFortessa, and FACSCanto‐LSRFortessa deviation in MFI from the reference instrument were calculated using the provided formula (Table 1). The defined acceptability criterion of <15% variation in MFI was met between synchronized FACSCanto II instruments (Figure 2A). Variation in MFI between different LSRFortessa instruments was also observed to be within the acceptable comparability criteria^3^ (Figure 2B). However, when comparing synchronized FACSCanto‐LSRFortessa variation in MFI, variation of five out of eight PMTs was widely out of the acceptable range (Table 2; Figure 2C).

**TABLE 1:** Deviation from reference MFI per fluorochrome after multicolor hard‐dyed bead synchronization of two FACSCanto II and two LSRFortessa flow cytometers

	Fluorochromes							
	FITC	PE	PerCP‐Cy5.5	PE‐Cy7	APC	APC‐A750	PB	PO
Evaluated antibody conjugates	CD2	CD7	CD3	CD14	CD8	CD19	CD4	CD45
FACSCanto II 1 vs FACSCanto II 2								
Mean reference MFI	10 965	4471	8240	6678	10 970	4546	3998	6768
Mean matched MFI	11 591	4600	8209	6695	10 154	3989	4079	6501
MFI difference	627	129	30	17	816	557	81	268
Mean % dev. from ref.	5.4%	2.8%	0.4%	0.3%	8.0%	14.0%	2.0%	4.1%
±SD	±0.7%	±2.0%	±1.0%	±1.1%	±2.9%	±1.2%	±4.1%	±2.7%
LSRFortessa 1 vs LSRFortessa 2								
Mean reference MFI	16 915	10 444	12 803	15 102	34 846	5263	8629	13 738
Mean matched MFI	15 990	10 346	12 936	15 101	33 497	5630	8324	13 429
MFI difference	924	98	133	1	1349	367	305	309
Mean % dev. from ref.	5.8%	0.9%	1.0%	0.0%	4.0%	6.5%	3.7%	2.3%
±SD	±7.6%	±0.8%	±1.5%	±1.5%	±1.7%	±4.3%	5.0%	±3.6%

Note

Mean reference MFI, mean matched MFI, the observed MFI difference, and percentage of deviation from reference MFI ± SD are shown when comparing two FACSCanto II (top) and two LSRFortessa flow cytometers (bottom). Data reflect results from at least six 8‐parameter stained whole‐blood samples after synchronization using the multicolor hard‐dyed beads. FACSCanto II vs FACSCanto II: n = 10; LSRFortessa vs LSRFortessa: n = 6.

Abbreviations: dev. from ref., deviation from reference; MFI, mean fluorescence intensity; SD, standard deviation.

**TABLE 2:** Deviation from reference MFI per fluorochrome after multicolor hard‐dyed or single‐fluorochrome–conjugated surface‐dyed bead synchronization of a FACSCanto and a LSRFortessa flow cytometer

	Fluorochromes							
	FITC	PE	PerCP‐Cy5.5	PE‐Cy7	APC	APC‐A750	PB	PO
Evaluated antibody conjugates	CD2	CD7	CD3	CD14	CD8	CD19	CD4	CD45
Hard‐dyed beads								
FACSCanto II vs LSRFortessa								
Mean reference MFI	9490	23 069	7197	7643	11 532	2837	7341	11 734
Mean matched MFI	8463	16 350	7146	12 810	19 000	2565	4675	8955
MFI difference	1027	6719	51	5167	7467	273	2666	2779
Mean % dev. from ref.	12.1%	41.1%	0.7%	40.3%	39.3%	10.6%	57.0%	31.0%
±SD	±9.4%	±8.6%	±2.3%	±1.9%	3.2%	±3.6%	±7.8%	±1.5%
Surface‐dyed beads								
FACSCanto II vs LSRFortessa								
Mean reference MFI	15 445	4437	4410	18 324	20 110	2587	5888	6164
Mean matched MFI	15 014	4699	4602	17 994	19 448	2702	6662	5542
MFI difference	432	262	192	331	662	116	774	622
Mean % dev. from ref.	2.9%	5.6%	4.2%	1.8%	3.4%	4.3%	11.6%	11.2%
±SD	±1.7%	±5.0%	±2.2%	±1.9%	±2.1%	±6.1%	2.2%	±2.4%

Note

Mean reference MFI, mean matched MFI, the observed MFI difference, and percentage of deviation from reference MFI ± SD are shown when utilizing the multicolor hard‐dyed bead (top) and single‐fluorochrome–conjugated surface‐dyed bead synchronization protocol (bottom). Data reflect results from five 8‐parameter stained whole‐blood samples.

Abbreviations: dev. from ref., deviation from reference; MFI, mean fluorescence intensity; SD, standard deviation.

### 3.2 Utilizing single‐fluorochrome–conjugated surface‐dyed beads for effective synchronization between the FACSCanto II and LSRFortessa

As multicolor hard‐dyed bead synchronization was found to be ineffective in synchronizing FACSCanto II and LSRFortessa instruments, the above‐described single‐fluorochrome–conjugated surface‐dyed bead synchronizing protocol was subsequently tested (Figure 1B). Compensation of spectral overlap was applied as described. Subsequently, five different eight‐parameter stained whole‐blood samples were analyzed on all flow cytometers, and variation was compared between FACSCanto II and LSRFortessa instruments (Table 2). Utilizing the single‐fluorochrome–conjugated surface‐dyed bead synchronization protocol at least halved the variations observed with the multicolor bead protocol. Variation in MFI between all parameters met the acceptable comparability criteria, indicating that the single‐fluorochrome bead protocol is a good alternative for the multicolor bead protocol for synchronization between FACSCanto II and LSRFortessa instruments (Figure 2D).

## 4 DISCUSSION AND CONCLUSION

Standardization of immunophenotyping to provide information for diagnosis and treatment of, for instance, neoplastic hematopoietic cells is crucial to avoid inconsistencies between clinical diagnostic laboratories. Excellent recommendations and guidelines have been reported to deal with standardization of sample preparations and synchronization of flow cytometer instruments.^3^, ^4^, ^5^, ^6^, ^7^, ^8^, ^13^, ^14^ However, as technology evolved, several new instruments have emerged which are equipped with a four (or even more)‐laser‐line configuration. In this study, we show that the defined acceptable comparability criteria (<15% variation in MFIs from the reference instrument) could be met when utilizing single‐fluorochrome–conjugated surface‐dyed beads to determine and adjust PMT voltages to synchronize our FACSCanto and LSRFortessa instruments. In contrast, defined comparability criteria could not be met when utilizing multicolor hard‐dyed beads for instrument synchronization.

In principle, all instruments containing a 405‐nm, 488‐nm, and 633‐ to 640‐nm excitation laser and at least two, four, and two detectors for each excitation line, respectively, fulfill the technical requirements for acquisition of the eight‐color panel of fluorochromes.^16^ Differences in laser power between instruments should be taken into account, as this causes differences in spread of the negative peaks and is independent of the utilized synchronization protocol.

Solly et al^9^ reported that multicolor hard‐dyed bead synchronization between FACSCanto II and Navios is feasible, but less effective compared to synchronization of instruments from the same manufacturer. Nováková et al^16^ shed light on the fact that synchronization of instruments from different manufacturers is hampered by adjusted emission filters for optimal detection of the manufacturers' proprietary fluorochromes. They report that extension of the EuroFlow SOP with single‐fluorochrome–conjugated surface‐dyed BD™ CompBeads to further synchronize PMT voltages between Navios, MACSQuant, and FACSCanto is necessary to meet the acceptable comparability criteria between these instruments. Blanco *et al* reported in the same issue that these settings can also be utilized to synchronize FACSCanto II and LSRFortessa instruments,^17^ even though hampered multicolor hard‐dyed bead synchronization cannot be explained by emission filter differences and is most probably due to the higher laser power of the LSRFortessa.

Hard‐dyed beads have incorporated surrogate dyes in their polymer matrix, causing them to merely share optical properties, but no spectral equivalence to the fluorochromes utilized in immunophenotyping of biological samples. Furthermore, incorporation of the dyes in the polymer matrix does not resemble fluorochrome‐stained biological samples. This is a major drawback of hard‐dyed bead–based synchronization, as synchronization of these internal surrogate dyes does not necessarily mean synchronization of the actual fluorochromes of interest.^15^ We therefore hypothesize that the differences in laser‐line configurations and laser power between FACSCanto II and LSRFortessa may result in different proportions between the surrogate dyes and the actual fluorochromes to be synchronized, causing differences in MFIs to occur when surrogate dye MFIs are matched. This is further substantiated by the fact that we are able to effectively synchronize FACSCanto II and LSRFortessa instruments when utilizing single‐fluorochrome–conjugated surface‐dyed beads.
